# Applicability of the User Engagement Scale to Mobile Health: A Survey-Based Quantitative Study

**DOI:** 10.2196/13244

**Published:** 2020-01-03

**Authors:** Marianne Holdener, Alain Gut, Alfred Angerer

**Affiliations:** 1 Winterthur Institute of Health Economics School of Management and Law Zurich University of Applied Sciences Winterthur Switzerland; 2 IBM Switzerland Ltd Zurich Switzerland

**Keywords:** mobile health, mhealth, mobile apps, user engagement, measurement, user engagement scale, chatbot

## Abstract

**Background:**

There has recently been exponential growth in the development and use of health apps on mobile phones. As with most mobile apps, however, the majority of users abandon them quickly and after minimal use. One of the most critical factors for the success of a health app is how to support users’ commitment to their health. Despite increased interest from researchers in mobile health, few studies have examined the measurement of user engagement with health apps.

**Objective:**

User engagement is a multidimensional, complex phenomenon. The aim of this study was to understand the concept of user engagement and, in particular, to demonstrate the applicability of a user engagement scale (UES) to mobile health apps.

**Methods:**

To determine the measurability of user engagement in a mobile health context, a UES was employed, which is a psychometric tool to measure user engagement with a digital system. This was adapted to Ada, developed by Ada Health, an artificial intelligence–powered personalized health guide that helps people understand their health. A principal component analysis (PCA) with varimax rotation was conducted on 30 items. In addition, sum scores as means of each subscale were calculated.

**Results:**

Survey data from 73 Ada users were analyzed. PCA was determined to be suitable, as verified by the sampling adequacy of Kaiser-Meyer-Olkin=0.858, a significant Bartlett test of sphericity (χ^2^_300_=1127.1; *P*<.001), and communalities mostly within the 0.7 range. Although 5 items had to be removed because of low factor loadings, the results of the remaining 25 items revealed 4 attributes: perceived usability, aesthetic appeal, reward, and focused attention. Ada users showed the highest engagement level with perceived usability, with a value of 294, followed by aesthetic appeal, reward, and focused attention.

**Conclusions:**

Although the UES was deployed in German and adapted to another digital domain, PCA yielded consistent subscales and a 4-factor structure. This indicates that user engagement with health apps can be assessed with the German version of the UES. These results can benefit related mobile health app engagement research and may be of importance to marketers and app developers.

## Introduction

### Background

In recent years, mobile apps addressing health and fitness issues have grown at a remarkable rate. Although there are significant advantages to health apps, such as lower health care costs and increased accessibility to health advice [1], their full potential is still untapped, mainly because of a failure to engage users in terms of sufficient and effective use [2,3].

User engagement has been recognized as a key factor in determining the success of an app [4-6] because it is linked to the user’s intention to continue using a mobile app [7,8]. Continued use, a subset of behavioral engagement, is a critical issue because of the highly competitive nature of the mobile app market [8,9]. However, it is important for researchers to move beyond user continuance behavior and examine user engagement as a broader concept [10].

Originally influenced by the user experience movement in human-computer interaction (HCI), user engagement has become a buzzword in various areas, including mobile health apps [11]. However, there is no general agreement as to what constitutes user engagement or how it is operationalized and measured [11,12].

### Prior Research

#### Theoretical Perspectives

Although there is no user engagement theory as such, some related theories provide insights into why users engage with technology [11]. In the absence of a specific theory, researchers have relied on established theories from other disciplines [13]. Much research on user engagement has been based on Csikszentmihalyi’s theory of optimal experiences, which is also known as the flow theory [11]. Particularly in the HCI and computer science literature, engagement is often seen as a subjective experience of flow [12]. Some of the attributes of flow, such as focused attention, feedback, control, and activity orientation, also occur in engagement [14]. However, it is suggested that the “degree and manifestation of these attributes may be what sets these concepts apart” (p.12) [11]. A further difference between user engagement and flow is how the 2 concepts are related to positive and negative emotions. In the flow theory, positive experience is particularly important [15], whereas user engagement is held to entail a more complex spectrum of emotions [11].

User engagement studies have also incorporated insights based on Dewey’s philosophy of experience [16]. Originally, Dewey aligned his philosophy of experience to the field of education. However, his views have also been perceived as convincing and helpful in clarifying aspects of experience in the HCI world. In particular, 4 threads of experience, rooted in Dewey’s philosophy, describe major aspects of users’ experience with technology, which are the sensual, emotional, compositional, and spatiotemporal threads [17]. The sensual thread affects sensory engagement, the emotional thread is concerned with how users engage with the product emotionally, the compositional thread refers to users’ relationships and interactions with others or with things, and the spatiotemporal thread is the aspect of space and time during the experience [17].

#### Concept of User Engagement

Without a doubt, engagement with technology is a multifaceted, inherently complex phenomenon [5,18,19]. Definitions of user engagement vary depending on the applications, settings, and variables of interest of user engagement research [5,13]. One of the earliest definitions proposed that “user engagement is a user’s response to an interaction that gains, maintains and encourages their attention, particularly when they are intrinsically motivated” [20]. Further definitions are given by various authors (eg, [9,18-22]), and a certain overlap of aspects is apparent. For the purpose of this study, the following commonly cited principles were considered. User engagement is a quality of user experience [21,22] and is multidimensional [9,18,22]. There is no consensus concerning the meaning of the dimensions, but the cognitive, emotional, and behavioral dimensions are prevalent in user engagement literature [11,14,23]. In addition, engagement can be viewed as a process during an interaction or as a product of experience [11,14,24]. This study focused on the product- or outcome-based view where engagement attributes are crucial because they represent what a user finds naturally compelling when interacting with technology [14].

#### Measurement of User Engagement

Its complex nature and varying definitions make user engagement difficult to quantify [13]. We used the search terms (“user engagement” OR “consumer engagement” OR “engagement”) AND (“mobile health*” OR “mobile health app*” OR “mHealth”) and queried the databases PubMed, Web of Science Core Collection, and ProQuest. This search determined 3 groups of user engagement measures: self-reported methods, physiological methods such as eye tracking, and user analytics methods such as dwell time. These methods and measures are often used in combination [13].

Self-reported measures are predominant in user engagement research because they are “useful for capturing users’ attitudes toward, cognitive appraisals of, and emotions surrounding their experiences of engagement with technology” (p.15) [24].

Self-reported measures target subjective user engagement by way of users’ perception of technology [24], mainly through postexperience questionnaires [13,24] to measure engagement characteristics based on an interactive experience in surveys conducted with large numbers of users [25].

We found 3 studies that explicitly stated to have examined the concept of user engagement within a health app: 2 of them operationalized and measured user engagement by means of metrics [26,27] and 1 used a mixed methods approach by analyzing app usage data and conducting a satisfaction survey [5]. Other studies were conducted in similar topic areas of user engagement, for example, user experience [28] or continued intention to use [29-31].

#### Research Problem and Question

The issue of user engagement is a concern for providers of health apps, as insufficient engagement by users with an app affects its success rate [32,33]. It has been reported that health app users are usually not very committed to a particular app and will use it only for a short time and in a casual manner [34]. This phenomenon is known as the law of attrition and describes the situation that in any electronic health (eHealth) trial, a substantial proportion of users drop out prematurely or stop using the app [35]. For example, a retrospective cohort study of a dietary self-monitoring mobile app discovered that only 3% of 190,000 downloads resulted in a person using the mobile food journal for more than 1 week [29]. This implies that high dropout rates are natural and are a typical feature of health apps. However, study results on the reasons for this phenomenon vary and mostly only identify the characteristics of health app users [36,37].

From an academic research perspective, there has been increasing interest from researchers in mobile health because of a growing number of health apps available. Past studies in this field can be divided into acceptance studies, design studies, and behavior change studies [1]. Nonetheless, research on the usage of health apps is still in its early stages [38].

In particular, too little attention has been paid to the conceptualization and measurement of user engagement with health apps [5,39]. Future research suggestions include features that might affect user acceptability and preferences [34], motivational factors that may lead to more sustained app usage [38], or other factors that relate to increased user engagement with commercially available health apps [26]. Furthermore, although user engagement has been conceptualized differently across the literature [11,12], a better understanding is required as to how different attributes of mobile apps influence user engagement [8]. Researchers agree that user engagement can best be operationalized by examining user system attributes that reflect an engaging experience and, therefore, constitute defining features of user engagement [24,40].

Considering the aforementioned gaps, this study sought to answer the following research question: to what extent does an existing user engagement scale (UES) yield consistent subscales in the context of health apps? As user engagement is context specific [11], for example, to the situation that the interaction triggers, and hence serves the purpose of collecting comparable data, the health app Ada is used as an example in this study. Ada was developed by Ada Health, a medical technology company based in Berlin, as a personal health guide that supports the user’s health care journey with a personalized interactive chat function [41]. In 2017, this medical app had the fastest growing number of users in Europe and was ranked as the number one medical app in 130 countries worldwide [42]. Furthermore, Ada’s chief executive officer has high ambitions: he and his team aim to achieve 100 million users by 2020 [42].

## Methods

### Scale

To answer the aforementioned research question in the best possible way, we first need to consider the scale. The recommendation when applying a self-reported measurement is to rely on previous questionnaires [24]. In the context of digital health, an eHealth engagement scale was developed [43]. We chose the eHealth scale because its characteristics are similar to ours. Both studies have an HCI setting [43], but they measured engagement with eHealth content on a website and in a laboratory context. The mobile aspect of our study is probably not as important because spatial data do not matter in both contexts. However, we still propose that through the smaller window of a phone, user engagement might be different. Therefore, this scale was not found to be an appropriate fit for this research context also because no other studies could be found that utilized this scale, causing us to question its robustness and validity.

A better fit was the UES by O’Brien et al [40], a psychometric tool used to measure user engagement with a digital system. Using empirical observations and theoretical elements of the flow theory, as well as John Dewey’s philosophy of experience, the original scale has in recent years been applied to over 40 published studies in various HCI settings, such as Web news, educational technologies, social networking, or information search [13]. Few studies have used this scale in its entirety, probably because of its length and insufficient data on how to administer it; moreover, the 6-factored solution was questioned in various studies [44,45]. As a consequence, another study conducted in 2018 presented a refined scale consisting of 30 items intended to measure the 4 dimensions of user engagement in HCI settings. Table 1 describes the 4 dimensions (based on [14,19,38)]. Researchers may use only subscales of the scale; however, user engagement as a holistic construct can then not be measured [40].

**Table 1:** User engagement scale dimensions.

Dimension	Description
Focused attention	On the basis of some characteristics of the flow theory: focused concentration, absorption, and temporal dissociation
Perceived usability	Affective (frustration) and cognitive (effortful) aspects as a result of the interaction
Aesthetic appeal	Sensory and visual appearance of an interface
Reward	Hedonic aspects of experience, felt involvement, overall success of the interaction, and willingness to engage with the app in the future

Therefore, we proposed the following hypothesis for our study:

H1: The UES can be used to assess user engagement with health apps.

### Data Collection

Using the full-length UES proposed by O’Brien et al [40], we designed an online survey using EFS Survey, a software package created by Questback. We had to make 1 major adaptation to the existing UES: a translation of the questions from English to German as we conducted the survey in German-speaking countries. Not all items are suitable or compatible in another language [40]. Nonetheless, we did not make any item selections before data collection; we translated and included all the items. We slightly modified some of the wording to adapt them to the context of Ada.

We pretested the online survey under field conditions on 4 participants, using the available pretest feature of the EFS Survey. This allows pretest participants to attach comments to individual questions. After the pretest, small changes and refinements to the wording and layout were made. In addition, the data export feature was tested to confirm that the collected data could be exported.

Data collection took place for 2 weeks in April 2018. A convenience sample as a sampling type was used. Accordingly, the link to the online survey was distributed to selected people. In addition, the snowball system was applied, whereby the participants were asked to share the survey link with their circle of friends and acquaintances. We asked participants to first download the health app Ada, use it at least once, and then relate the questions to their experience with Ada in completing the survey. We assessed the survey questions using 5-point Likert scales. The 30 questions of the UES were randomized, and information about user engagement dimensions was hidden. The English items and their corresponding translations into German can be found in Multimedia Appendix 1.

### Data Analysis

When using the UES, it is suggested to perform factor analysis [40]. As the aims of this study were to understand the concept of user engagement and test the applicability of the UES to a mobile health app, a principal component analysis (PCA) with varimax rotation was performed. The main reason for choosing PCA instead of factor analysis was that the UES had originally been developed in English and that there is no German version to date.

Before conducting the main analysis, we considered several conceptual and statistical issues. These initial evaluations are of high importance because of the dependence of the quality of the data on the results of factor analysis [46]. We examined the sample size, communalities, and correlations between the items. In addition, the Kaiser-Meyer-Olkin (KMO) measure and Bartlett test of sphericity were considered.

Once this is checked, the number of extracting factors can be determined. All 3 criteria (scree test criterion, Kaiser criterion, and an a priori criterion) were discussed. Next, the factor structure was evaluated. To do so, the values of the factor loadings, which is the correlation between the original variable and its factor, were considered [47,48]. We followed the recommendation by Backhaus et al [46] and only assigned variables with loadings higher than 0.5.

After the factor structure had been defined, sum scores for each subscale were calculated. Sum scores are calculated as means for each subscale [40]. As some of the variables were reverse phrased (v_08, v_10-v_13, and v_23; see Multimedia Appendix 1), they were first recoded and transferred to the same variable so that values could be compared. Furthermore, because of the multidimensionality character of the UES, reliability in the form of internal consistency of the subscales was examined separately for each subscale using Cronbach alpha. Statistical data analysis was conducted using the statistics program SPSS version 23 (IBM).

## Results

### Participants and Descriptive Statistics

In total, the survey link was viewed 363 times, which translated to 73 responses. As all the questions were programmed as mandatory questions, there are no missing values; thus, none of the participants needed to be excluded from further analysis. Out of 73 participants, 36 (49%) were female and 37 (51%) were male. Their average age was 39 years (SD 15.4 years), with the youngest participant being 18 years and the oldest participant being 73 years. Multimedia Appendix 2 contains a summary of participant demographics.

### Principal Component Analysis

A PCA was conducted on the 30 items using varimax rotation. PCA was determined to be suitable, as verified by multiple criteria. First, the sample size of this study, 73, was above the minimum absolute sample size of 50 [48]. Second, there were correlations below 0.3. It is recommended to exclude variables that correlate below 0.3 or correlate above 0.9 with any other variable [47,49]. However, as no variables had zero correlations below 0.3, all variables were retained for further analysis. Furthermore, there were no correlations above 0.9. Third, communalities were mostly within the 0.7 range. With a sample size of 73, communalities of around 0.7 were deemed sufficient [49]. In addition, the factorability of the items was verified by the sampling adequacy of KMO=0.858 (*meritious* according to Kaiser and Rice [50]) and a significant Bartlett test of sphericity (*χ^2^*_300_=1127.1; *P*<.001). In conclusion, based on the consideration of these criteria, factor analysis was determined to be suitable.

The next step was to obtain the factors. Overall, 4 factors had eigenvalues over Kaiser criterion of 1 and, in combination, explained 65.1% of the variance. The scree plot was ambiguous and showed inflections that would justify retaining both 2 and 4 factors. The a priori criterion indicated 4 factors [40]. Thus, 4 factors were retained because they accord with Kaiser criterion, scree plot, and the a priori criterion.

Furthermore, 5 items showed factor loadings below 0.5 (v_07, v_09, v_14, v_15, and v_26) and were therefore removed from PCA as their correlation with other variables was not strong enough. One variable, v_16, showed cross-loadings, as it loaded on factor 1 (0.533) and on factor 3 (0.582). Such variables either need to be excluded or interpreted with both factors, unless strong theoretical reasons speak against this [46]. In this case, this variable was theoretically expected to load on factor 3, and indeed, the higher factor loading of this variable was on factor 3. This variable was, therefore, kept and assigned to factor 3.

The results of the PCA are illustrated in Multimedia Appendix 3, in which the variables are listed within the respective factors in a descending order. Overall, this PCA yielded consistent subscales and a 4-factor solution as suggested by O’Brien et al [40]. Therefore, the factor labels were taken over. Only 1 of the 25 items, v_29, loaded on another factor (focused attention), as suggested by the original UES.

Factor 1, focused attention, accounted for 34% of the variance and consisted of items v_01-06 and v_29. This factor was the original UES’s focused attention subscale, with the addition of 1 reward element, v_29. Factor 2, perceived usability, accounted for 14% of the variance and consisted of items v_08 and v_10-13. Factor 3, aesthetic appeal, accounted for 9% of total variance and consisted of item v_16-20. This factor was the original aesthetics subscale. Factor 4, reward, accounted for 8% of variance and consisted of v_21-25, v_27-28, and v_30.

### Sum Scales

In addition, sum scores were calculated for each factor. The factor perceived usability had the highest sum, with a value of 294; second was the factor aesthetic appeal, with a value of 275.4; third was the factor reward, with a value of 259.5; and the lowest subscale score was for the factor focused attention, with a value of 198.1. In other words, users of Ada showed the highest engagement level with perceived usability and the lowest engagement level with focused attention.

### Post Analysis

Focused attention, aesthetic appeal, and reward subscale of the UES all had high reliabilities, with Cronbach alpha=.912, alpha=.852, and alpha=.910, respectively. However, the perceived usability subscale had a lower reliably, with Cronbach alpha=.693 (see Multimedia Appendix 4).

## Discussion

### Principal Findings

The results of this study confirm the proposed hypothesis by demonstrating that the UES developed for measuring user engagement with digital technology can be used to assess user engagement with health apps. We had to remove 5 items because of factor loadings below 0.5. Possible reasons for removing these factors are suggested in Multimedia Appendix 5. The modified 25-item version of the scale accounted for 65% of the variance in user engagement. Overall, 4 factors emerged: focused attention, perceived usability, aesthetic appeal, and reward.

In O’Brien et al [40]’s and our study, all items loaded on the same factors except for v_29 (“I felt involved in this experience”). In our study, it loaded on focused attention rather than reward. This can be explained by taking into consideration our research context of health apps and the flow theory. This item indicates absorption in the experience with Ada, and absorption is a major characteristic of the flow theory, therefore making it a good fit for the focused attention factor. Aside from this, the suggested factor structure could be replicated in this study.

### Limitations

Although our findings support the use of the German UES with health apps, this study is subject to certain limitations, and results should be interpreted with caution.

First, data were collected from a sample that was not selected randomly, and the characteristics of the population were not adequately represented in the sample. In addition, the number of participants, 73, is rather small, although it is sufficient for conducting factor analysis [48].

Second, the UES was administrated and adapted to the context of this study. All items were taken from English and translated by the authors. Therefore, some items may have a slightly different meaning to respondents than the items in the original instrument. In addition, some of the items did not make sense in a non-English context and had to be removed because of low factor loadings during analysis.

The third limitation concerns the UES itself. In this study, no items were added to the original scale, so construct validity is not threatened. However, this tool might not capture all the important determinants of user engagement. In addition, adding another research method, for example, interviewing the participants, would have contributed to the interpretation of our findings.

Fourth, engagement was only measured once during a short period and not across multiple sessions, which may represent a limited view on user engagement. Further research could examine user engagement by having participants complete the scale more than once as part of the same study. Researchers could then compare engagement among participants and between iterations [40] and take a long-term view on engagement.

Furthermore, the question arises if participants engaged fully and intrinsically with the health app by being initially asked to use it at least once. Other user engagement studies in different fields have had similar problems (eg, [43,51]). Future research should try to address this issue.

### Comparison With Prior Work

This study is among the first to investigate the use of the UES as a whole in the area of health apps and in the German language, so the findings cannot be compared with those of other studies. However, patterns of the UES may be compared with those of other app user engagement studies. Still, direct comparison of attributes and factors within the area of health app user engagement has to be treated with caution because of the different approaches used. This, finally, comes back to various perceptions of what constitutes user engagement and what does not.

Furthermore, the high context dependence of the defining features of user engagement are reported [11], and in the context of mobile apps, it is probably also a question of the mobile app type examined. For example, mobile app types might be divided into experiential and informational app types [52]. According to this distinction, Ada, the example of this study, falls into the category of informational apps and focuses on goal-oriented and utilitarian benefits rather than on social and hedonic benefits, as is the case for experiential app types. It has been discovered that the effect of time convenience on mobile app engagement is greater for informative mobile apps than for experiential apps [9]. Applying this to user engagement with Ada might lead to the conclusion that utilitarian benefits, such as time convenience, are better suited to explain the factor of reward than other mobile app types would have.

### Conclusions

User engagement is a complex concept, and there is no general agreement as to what constitutes the phenomenon or how it is operationalized and measured [11,12]. This paper contributes in several ways to the growing literature concerning user engagement with health apps.

One major contribution of this study is the applicability of a German version of the UES [40] with a health app. On the basis of our findings, researchers and practitioners can now further investigate the user engagement concept. Future research could build on our findings and interpret the data gathered with the UES by applying a multiple method design. Owing to its scope and complexity, researchers have taken a multidimensional view of user engagement [5,13]. In addition, further research could investigate the use of the UES with apps in general and not be limited to health apps.

Given the importance of user engagement, the findings of this study could benefit practitioners, mainly marketers and app developers, in 2 ways.

First, marketers and app developers can gain a better understanding of user engagement. Evidence suggests that one of the main challenges for successful companies in mobile environments is to find ways to keep their users engaged [4-6]. Knowing what keeps users engaged has important implications for strategic retention management [9].

Second, findings based on the self-reported measurement of the German version of the UES with Ada suggest that the attribute that drives the highest engagement among users is perceived usability. This is followed by aesthetic appeal, reward, and focused attention.

An improved understanding of the attributes that drive user engagement should help app developers and marketers in creating and marketing attractive health care apps that will be used and appreciated in the long term. This could be a vital factor in increasing health literacy among users and, therefore, a contribution toward improving public health in general.

## Appendix

Multimedia Appendix 1 User engagement scale, German version.

Multimedia Appendix 2 Participant demographics.

Multimedia Appendix 3 Summary of principal component analysis results for the user engagement scale, German version (N=73).

Multimedia Appendix 4 Reliability analysis of user engagement scale dimensions.

Multimedia Appendix 5 Explanation of removed items.

## Abbreviations

eHealth: electronic health
HCI: human-computer interaction
KMO: Kaiser-Meyer-Olkin
PCA: principal component analysis
UES: user engagement scale

## References

[1] Sahin C (2018). Rules of engagement in mobile health: what does mobile health bring to research and theory?. Contemp Nurse.

[2] Kohl LF, Crutzen R, de Vries NK (2013). Online prevention aimed at lifestyle behaviors: a systematic review of reviews. J Med Internet Res.

[3] Nicholas J, Fogarty AS, Boydell K, Christensen H (2017). The reviews are in: a qualitative content analysis of consumer perspectives on apps for bipolar disorder. J Med Internet Res.

[4] Jones SP, Patel V, Saxena S, Radcliffe N, Ali Al-Marri S, Darzi A (2014). How Google's 'ten Things We Know To Be True' could guide the development of mental health mobile apps. Health Aff (Millwood).

[5] Taki S, Lymer S, Russell CG, Campbell K, Laws R, Ong K, Elliott R, Denney-Wilson E (2017). Assessing user engagement of an mHealth intervention: development and implementation of the growing healthy app engagement index. JMIR Mhealth Uhealth.

[6] Zhao Z, Balagué C (2015). Designing branded mobile apps: fundamentals and recommendations. Bus Horiz.

[7] Kim YH, Kim DJ, Wachter K (2013). A study of mobile user engagement (MoEN): engagement motivations, perceived value, satisfaction, and continued engagement intention. Decis Support Syst.

[8] Tarute A, Nikou S, Gatautis R (2017). Mobile application driven consumer engagement. Telemat Inform.

[9] Kim S, Baek T (2018). Examining the antecedents and consequences of mobile app engagement. Telemat Inform.

[10] Fang J, Zhao Z, Wen C, Wang R (2017). Design and performance attributes driving mobile travel application engagement. Int J Inf Manag.

[11] O'Brien HL, O'Brien HL, Cairns P (2016). Theoretical perspectives on user engagement. Why Engagement Matters: Cross-Disciplinary Perspectives of User Engagement in Digital Media.

[12] Perski O, Blandford A, West R, Michie S (2017). Conceptualising engagement with digital behaviour change interventions: a systematic review using principles from critical interpretive synthesis. Transl Behav Med.

[13] O'Brien HL, O'Brien HL, Cairns P (2016). Translating theory into methodological practice. Why Engagement Matters: Cross-Disciplinary Perspectives of User Engagement in Digital Media.

[14] O'Brien HL, Toms EG (2008). What is user engagement? A conceptual framework for defining user engagement with technology. J Am Soc Inf Sci Technol.

[15] Nakamura J, Csikszentmihalyi M, Snyder CR, Lopez SJ (2009). Flow theory and research. Oxford Handbook of Positive Psychology. Second Edition.

[16] Dewey J (1938). Experience And Education. First Touchstone edition 1997.

[17] Mccarthy J, Wright P (2004). Technology As Experience.

[18] Attfield S, Kazai G, Lalmas M, Piwowarski B (2011). Towards a Science of User Engagement. Paper presented at the WSDM workshop on user modelling for web applications.

[19] Sutcliffe A (2009). Designing for User Engagement: Aesthetic and Attractive User Interfaces.

[20] Jacques RD (1996). The Nature of Engagement and its Role in Hypermedia Evaluation and Design.

[21] O'Brien HL, Toms EG (2009). The development and evaluation of a survey to measure user engagement. J Am Soc Inf Sci Technol.

[22] O'Brien HL (2017). Antecedents and learning outcomes of online news engagement. J Assoc Inf Sci Technol.

[23] Dovaliene A, Masiulyte A, Piligrimiene Z (2015). The relations between customer engagement, perceived value and satisfaction: the case of mobile applications. Procedia Soc Behav Sci.

[24] Lalmas M, O'Brien H, Yom-Tov E (2014). Measuring User Engagement. Synth Lec Inf Concept Retrieval Serv.

[25] Lalmas M, O'Brien H, Yom-Tov E (2013). Measuring User Engagement. Proceedings of the 2013 World Wide Web Conference.

[26] Serrano KJ, Coa KI, Yu M, Wolff-Hughes DL, Atienza AA (2017). Characterizing user engagement with health app data: a data mining approach. Transl Behav Med.

[27] Rahman QA, Janmohamed T, Pirbaglou M, Ritvo P, Heffernan JM, Clarke H, Katz J (2017). Patterns of user engagement with the mobile app, manage my pain: results of a data mining investigation. JMIR Mhealth Uhealth.

[28] Anderson K, Burford O, Emmerton L (2016). Mobile health apps to facilitate self-care: a qualitative study of user experiences. PLoS One.

[29] Helander E, Kaipainen K, Korhonen I, Wansink B (2014). Factors related to sustained use of a free mobile app for dietary self-monitoring with photography and peer feedback: retrospective cohort study. J Med Internet Res.

[30] Cho J (2016). The impact of post-adoption beliefs on the continued use of health apps. Int J Med Inform.

[31] Lee HE, Cho J (2017). What motivates users to continue using diet and fitness apps? Application of the uses and gratifications approach. Health Commun.

[32] Laing BY, Mangione CM, Tseng C, Leng M, Vaisberg E, Mahida M, Bholat M, Glazier E, Morisky DE, Bell DS (2014). Effectiveness of a smartphone application for weight loss compared with usual care in overweight primary care patients: a randomized, controlled trial. Ann Intern Med.

[33] Becker S, Miron-Shatz T, Schumacher N, Krocza J, Diamantidis C, Albrecht U (2014). mHealth 2.0: experiences, possibilities, and perspectives. JMIR Mhealth Uhealth.

[34] Dennison L, Morrison L, Conway G, Yardley L (2013). Opportunities and challenges for smartphone applications in supporting health behavior change: qualitative study. J Med Internet Res.

[35] Eysenbach G (2005). The law of attrition. J Med Internet Res.

[36] Blanson HO, Rogers W, Dumay A (2011). Personal characteristics and the law of attrition in randomized controlled trials of eHealth services for self-care. Gerontology.

[37] Bol N, Helberger N, Weert Jcm (2018). Differences in mobile health app use: a source of new digital inequalities?. Inf Soc.

[38] Birkhoff SD, Smeltzer SC (2017). Perceptions of smartphone user-centered mobile health tracking apps across various chronic illness populations: an integrative review. J Nurs Scholarsh.

[39] Ubhi HK, Michie S, Kotz D, van Schayck OC, Selladurai A, West R (2016). Characterising smoking cessation smartphone applications in terms of behaviour change techniques, engagement and ease-of-use features. Transl Behav Med.

[40] O’Brien HL, Cairns P, Hall M (2018). A practical approach to measuring user engagement with the refined user engagement scale (UES) and new UES short form. Int J Hum Comput Stud.

[41] (2018). Ada.

[42] Nikolova S Research2Guidance.

[43] Lefebvre CR, Tada Y, Hilfiker SW, Baur C (2010). The assessment of user engagement with eHealth content: the eHealth engagement scale. J Comput Mediat Commun.

[44] O’Brien H, Cairns P (2015). An empirical evaluation of the User Engagement Scale (UES) in online news environments. Inf Process Manag.

[45] Wiebe EN, Lamb A, Hardy M, Sharek D (2014). Measuring engagement in video game-based environments: investigation of the user engagement scale. Comput Hum Behav.

[46] Backhaus K, Erichson B, Plinke W, Weiber R (2016). Multivariate Analysemethoden: Eine anwendungsorientierte Einführung. Fourteenth Edition.

[47] Field A (2018). Discovering Statistics using IBM SPSS Statistics. Fifth Edition.

[48] Hair JF, Black WC, Babin BJ, Anderson RE (2014). Multivariate Data Analysis. Seventh Edition.

[49] MacCallum RC, Widaman KF, Zhang S, Hong S (1999). Sample size in factor analysis. Psychol Method.

[50] Kaiser HF, Rice J (2016). Little Jiffy, Mark Iv. Educ Psychol Meas.

[51] O’Brien HL, Toms EG (2013). Examining the generalizability of the User Engagement Scale (UES) in exploratory search. Inf Process Manag.

[52] Bellman S, Potter RF, Treleaven-Hassard S, Robinson JA, Varan D (2011). The effectiveness of branded mobile phone apps. J Interact Market.

