# Supplementary material for: Applicability of the User Engagement Scale to Mobile Health: A Survey-Based Quantitative Study
Source: JMIR Mhealth Uhealth. 2020 Jan 3;8(1):e13244. doi: 10.2196/13244 (PMC6969386; doi:10.2196/13244)
Supplement: Multimedia Appendix 1 [file mhealth_v8i1e13244_app1.docx]

## Multimedia Appendix 1

|  | Item user engagement scale (based on [38]). | Item user engagement scale, German version |
| --- | --- | --- |
| **Dimension focused attention** | | |
| v_01 | I lost myself in this experience. | Ich habe mich bei dieser Anwendung vergessen. |
| v_02 | I was so involved in this experience that I lost track of time. | Ich war so in die Anwendung vertieft, dass ich die Zeit vergessen habe. |
| v_03 | I blocked out things around me when I was using Ada. | Ich blendete alles um mich herum aus, als ich Ada verwendete. |
| v_04 | When I was using Ada, I lost track of the world around me. | Als ich Ada verwendete, vergass ich alles um mich herum. |
| v_05 | The time I spent using Ada just slipped away. | Die Zeit verging wie im Flug, als ich Ada anwendete. |
| v_06 | I was absorbed in this experience. | Ich war gänzlich in die Anwendung vertieft. |
| **Dimension perceived usability** | | |
| v_07 | During this experience I let myself go. | Als ich Ada verwendete, konnte ich mich gehen lassen. |
| v_08 | I felt frustrated while using Ada. | Ich war frustriert, während ich Ada nutzte. |
| v_09 | I found Ada confusing to use. | Ich fand die Anwendung von Ada verwirrend. |
| v_10 | I felt annoyed while using Ada. | Ich fühlte mich genervt während der Verwendung von Ada. |
| v_11 | I felt discouraged while using Ada. | Ich fühlte mich entmutigt, während ich Ada nutzte. |
| v_12 | Using Ada was taxing. | Die Benutzung von Ada war anstrengend. |
| v_13 | This experience was demanding. | Diese Erfahrung war anspruchsvoll. |
| v_14 | I felt in control while using Ada. | Ich hatte die Kontrolle über Ada. |
| v_15 | I could not do some of the things I needed to do while using Ada. | Ich konnte nicht alle Anwendungen ausführen, die ich mit Ada ausführen wollte. |
| **Dimension aesthetic appeal** | | |
| v_16 | Ada was attractive. | Ada war attraktiv. |
| v_17 | Ada was aesthetically appealing. | Ada war ästhetisch ansprechend. |
| v_18 | I liked the graphics and images of Ada. | Mir gefielen die Grafiken und Bilder von Ada. |
| v_19 | Ada appealed to the visual senses. | Ada spricht auf visuelle Sinne an. |
| v_20 | The screen layout of Ada was visually pleasing. | Das Bildschirmlayout von Ada war optisch ansprechend. |
| **Dimension reward** | | |
| v_21 | Using Ada was worthwhile. | Die Nutzung von Ada hat sich gelohnt. |
| v_22 | I consider my experience a success. | Ich erachte meine Erfahrungen mit Ada als erfolgreich. |
| v_23 | This experience did not work out the way I had planned. | Die Erfahrung mit Ada hat nicht so funktioniert, wie ich es mir vorgestellt hatte. |
| v_24 | My experience was rewarding. | Meine Erfahrung mit Ada hat sich gelohnt. |
| v_25 | I would recommend Ada to my family and friends. | Ich würde Ada meiner Familie und meinen Freunden weiterempfehlen. |
| v_26 | I continued to use Ada out of curiosity. | Aus Neugierde habe ich Ada weitergenutzt. |
| v_27 | The content of Ada incited my curiosity. | Der Inhalt von Ada hat meine Neugier geweckt. |
| v_28 | I was really drawn into this experience. | Ich war wirklich von der Erfahrung mit Ada fasziniert. |
| v_29 | I felt involved in this experience. | Die Anwendung hat mich in ihren Bann gezogen. |
| v_30 | This experience was fun. | Diese Erfahrung hat Spass gemacht |
