# Supplementary material for: Applicability of the User Engagement Scale to Mobile Health: A Survey-Based Quantitative Study
Source: JMIR Mhealth Uhealth. 2020 Jan 3;8(1):e13244. doi: 10.2196/13244 (PMC6969386; doi:10.2196/13244)
Supplement: Multimedia Appendix 2 [file mhealth_v8i1e13244_app2.docx]

## Multimedia Appendix 2

| Characteristic | | n (%) |
| --- | --- | --- |
| **Total participants** | |  |
|  | Included | 73 (100%) |
|  | Excluded | 0 (0%) |
| **Gender** | |  |
|  | Female | 36 (51%) |
|  | Male | 37 (49%) |
| **Age (years)** | |  |
|  | 18-25 | 13 (18%) |
|  | 26-35 | 26 (35%) |
|  | 36-45 | 9 (12%) |
|  | 46-55 | 9 (12%) |
|  | 56-65 | 11 (15%) |
|  | >65 | 5 (7%) |
| **Country of residence** | |  |
|  | Switzerland | 72 (99%) |
|  | Germany | 1 (1%) |
|  | Austria | 0 (0%) |
