# Supplementary material for: Applicability of the User Engagement Scale to Mobile Health: A Survey-Based Quantitative Study
Source: JMIR Mhealth Uhealth. 2020 Jan 3;8(1):e13244. doi: 10.2196/13244 (PMC6969386; doi:10.2196/13244)
Supplement: Multimedia Appendix 3 [file mhealth_v8i1e13244_app3.docx]

## Multimedia Appendix 3

| **Variable** | **Focused attention** | **Perceived usability** | **Aesthetic appeal** | **Reward** |
| --- | --- | --- | --- | --- |
| v_02 | 0.891 |  |  |  |
| v_04 | 0.859 |  |  |  |
| v_03 | 0.807 |  |  |  |
| v_01 | 0.786 |  |  |  |
| v_29 | 0.729 |  |  |  |
| v_05 | 0.691 |  |  |  |
| v_06 | 0.679 |  |  |  |
| v_11 |  | 0.692 |  |  |
| v_12 |  | 0.686 |  |  |
| v_10 |  | 0.623 |  |  |
| v_13 |  | 0.597 |  |  |
| v_08 |  | 0.586 |  |  |
| v_20 |  |  | 0.869 |  |
| v_17 |  |  | 0.827 |  |
| v_18 |  |  | 0.736 |  |
| v_19 |  |  | 0.706 |  |
| v_16 |  |  | 0.582 |  |
| v_21 |  |  |  | 0.880 |
| v_24 |  |  |  | 0.835 |
| v_25 |  |  |  | 0.787 |
| v_22 |  |  |  | 0.782 |
| v_28 |  |  |  | 0.755 |
| v_27 |  |  |  | 0.733 |
| v_30 |  |  |  | 0.614 |
| v_23 |  |  |  | -0.604 |
| Eigenvalues | 8.611 | 3.596 | 2.137 | 1.942 |
| % of variance | 34.442 | 14.382 | 8.549 | 7.769 |
