# Supplementary material for: Applicability of the User Engagement Scale to Mobile Health: A Survey-Based Quantitative Study
Source: JMIR Mhealth Uhealth. 2020 Jan 3;8(1):e13244. doi: 10.2196/13244 (PMC6969386; doi:10.2196/13244)
Supplement: Multimedia Appendix 4 [file mhealth_v8i1e13244_app4.docx]

## Multimedia Appendix 4

| **Factor** | **Number items** | **Cronbach alpha** | **Mean (SD)** |
| --- | --- | --- | --- |
| Focused attention | 7 | .912 | 2.714 (0.909) |
| Perceived usability | 5 | .693 | 4.027 (0.638) |
| Aesthetic appeal | 5 | .852 | 3.773 (0.812) |
| Reward | 8 | .910 | 3.555 (0.857) |
