# Supplementary material for: Applicability of the User Engagement Scale to Mobile Health: A Survey-Based Quantitative Study
Source: JMIR Mhealth Uhealth. 2020 Jan 3;8(1):e13244. doi: 10.2196/13244 (PMC6969386; doi:10.2196/13244)
Supplement: Multimedia Appendix 5 [file mhealth_v8i1e13244_app5.docx]

## Multimedia Appendix 5

| **Removed Item English** | **Possible reason** |
| --- | --- |
| v_07 (During this experience I let myself go) | The English expression “I let myself go”, from which this item was translated, might be a commonly used expression in anglosphere countries, but in German this expression is not often encountered. A study applying the UES to exploratory search reported that even in English, this item was the one with the most missing values [36]. A possible reason for this might be that “this item uses a colloquial expression that may not have resonated with all participants” [36, p. 1098]. |
| v_09 (I found Ada confusing to use) | A reason for this item’s low loading might be found when analyzing user engagement with the app Ada. It is likely that few to no survey participants had previously used a health chatbot. Whether this referred to the use of Ada in terms of usability or to its content is unclear. |
| v_14 (I felt in control while using Ada) | Items v_14 and v_15 both correlated with only one other item of above 0.3. None of the other items showed only one correlation of above 0.3. Low factor loadings could be explained by the fact that participants of the survey were explicitly asked to try the app. Participants might, therefore, have used Ada because they were asked to do so, and not because they had a particular aim in mind. |
| v_15 (I could not do some of the things I needed to do while using Ada) | See reason above. |
| v_26 (I continued to use Ada out of curiosity) | Similarly, low factor loadings for v_26 may be explained by possible first-time usage of Ada. |
